# Quantitative Analysis of Surface Contouring with Pulsed Bipolar Radiofrequency on Thin Chondromalacic Cartilage

**DOI:** 10.1155/2020/1242086

**Published:** 2020-02-27

**Authors:** Michaela Huber, Daniela Schlosser, Susanne Stenzel, Johannes Maier, Girish Pattappa, Richard Kujat, Birgit Striegl, Denitsa Docheva

**Affiliations:** ^1^Department of Trauma Surgery & Emergency Department, University Medical Center Regensburg, Regensburg, Germany; ^2^Experimental Trauma Surgery, Department of Trauma Surgery, University Medical Center Regensburg, Regensburg, Germany; ^3^Regensburg Medical Image Computing (ReMIC), OTH Regensburg, Regensburg, Germany; ^4^Center of Biomedical Engineering, OTH Regensburg, Regensburg, Germany

## Abstract

The purpose of this study was to evaluate the quality of surface contouring of chondromalacic cartilage by bipolar radio frequency energy using different treatment patterns in an animal model, as well as examining the impact of the treatment onto chondrocyte viability by two different methods. Our experiments were conducted on 36 fresh osteochondral sections from the tibia plateau of slaughtered 6-month-old pigs, where the thickness of the cartilage is similar to that of human wrist cartilage. An area of 1 cm^2^ was first treated with emery paper to simulate the chondromalacic cartilage. Then, the treatment with RFE followed in 6 different patterns. The osteochondral sections were assessed for cellular viability (live/dead assay, caspase (cell apoptosis marker) staining, and quantitative analysed images obtained by fluorescent microscopy). For a quantitative characterization of none or treated cartilage surfaces, various roughness parameters were measured using confocal laser scanning microscopy (Olympus LEXT OLS 4000 3D). To describe the roughness, the Root-Mean-Square parameter (Sq) was calculated. A smoothing effect of the cartilage surface was detectable upon each pattern of RFE treatment. The Sq for native cartilage was Sq = 3.8 ± 1.1 *μ*m. The best smoothing pattern was seen for two RFE passes and a 2-second pulsed mode (B2p2) with an Sq = 27.3 ± 4.9 *μ*m. However, with increased smoothing, an augmentation in chondrocyte death up to 95% was detected. Using bipolar RFE treatment in arthroscopy for small joints like the wrist or MCP joints should be used with caution. In the case of chondroplasty, there is a high chance to destroy the joint cartilage.

## 1. Introduction

In recent years, treatment of cartilage degeneration with radiofrequency energy (RFE) remains controversial. Many experimental studies have shown that using RFE can lead to severe chondrocyte damage, if temperatures above 45°C are applied directly to the cartilage layer [1–3]. The chondrocyte death rate is proportional to the temperature increase. Edwards et al. reported a 40% chondrocyte death rate at a temperature of 55°C and almost 100% at 65°C [4]. The temperature elevation during an arthroscopic procedure is also time-dependent, as the longer the RFE electrode is activated, the higher is the temperature. However, several studies demonstrated that an activation of the energy flow between 3 and 10 seconds should be safe enough for use in arthroscopy [5, 6].

The positive effect of RFE compared to mechanical debriding is the “sealing effect” of the cartilage layer that stabilizes the damaged cartilage [7–12]. However, this “sealing effect” is also time-dependent. RFE of 15 seconds via a monopolar device resulted in a visibly smoother cartilage surface, as observed using electron microscopy, whilst a similar effect was also obtained with a bipolar device. However, in the latter case, a deeper chondrocyte damage was noted [13]. The above experiments were conducted on fresh osteochondral sections with chondromalacic cartilage from patients undergoing knee arthroplasty. An area of 1 cm^2^ was placed in a custom designed holder and treated with a meander-like pattern and cooled with a lavage fluid (22°C). In our opinion, these results cannot be compared to the impact of RFE in the arthroscopy of the wrist, due to the fact that the thickness of human knee cartilage is between 3 and 4 mm [14], whilst the cartilage layer in the wrist is between 0.7 and 1.2 mm [15]. Furthermore, a previous study has reported that a single RFE application of 2 seconds during wrist arthroscopy [5] reaches a mean temperature of around 24°C in the subchondral layer.

In our present study, we evaluated the smoothness of the cartilage surface generated by different RFE treatment patterns, as well as the vitality and apoptosis of the cartilage resident cells using a detailed quantitative analyses [16]. Based on the above literature evidence, we hypothesized that with a pulsed application of RFE, we can lower the apoptotic rate and increase chondrocytes' vitality caused by its continuous use with the concomitant rise in temperature.

## 2. Materials and Methods

### 2.1. Study Sample Preparation

**Figure 1:**
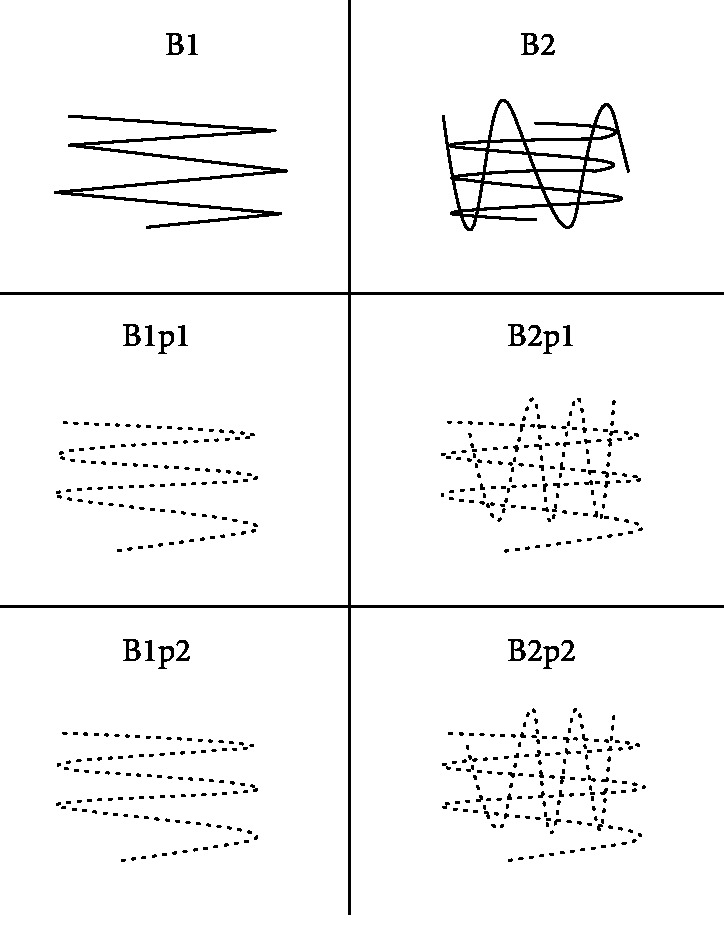
RFE treatment patterns used on chondromalacic cartilage with different treatment patterns: B1 = continuous treatment, 1 pass; B2 = continuous treatment, 2 passes; B1p1 = pulsed Treatment 1 second, 1 pass; B1p2 = pulsed Treatment 1 second, 2 passes; B1p2 = pulsed Treatment 2 seconds, 1 pass; and B2p2 = pulsed Treatment 2 seconds, 2 passes.

Knees where dissected from freshly slaughtered 6-month-old pigs, and 9 tibia plateaus were utilized for the experiments. The thickness of the porcine chondral layer is similar to that of the human radius cartilage with a mean thickness of 0.9 mm. Areas of 1 cm^2^ (4 independent times) were marked, and their middles had 1 mm subchondral holes drilled to fit a temperature sensor (platinum-chip-sensors, Pt 1000, TYP PCA, 1.1505.10 M JUMO, Fulda, Germany). Then, the marked areas were treated with a commercially available emery paper (size P60), using a manual grinding procedure, to simulate an outerbridge grade III osteoarthritis (OA), that was evaluated in a previous experiment [17]. In our previous experiment, the different roughness induced through the emery paper was compared with fresh osteochondral sections taken from hip arthroplasties, where the cartilage defect was graded according to the outerbridge classification. Then, the tibia plateaus were positioned in a custom-made holder, filled with 0.9% NaCl at room temperature and a flow rate of 50 ml/min with a gravity-assisted outflow was applied. Afterwards, the induced OA areas were treated with the bipolar radiofrequency electrode (RFE) (VAPR II 2.3 mm side effect, Depuy Mitek, Westwood, MA, USA) in an ablation mode using a non touch technique. Six different treatment patterns were applied as depicted in Figure 1.

**Figure 2:**
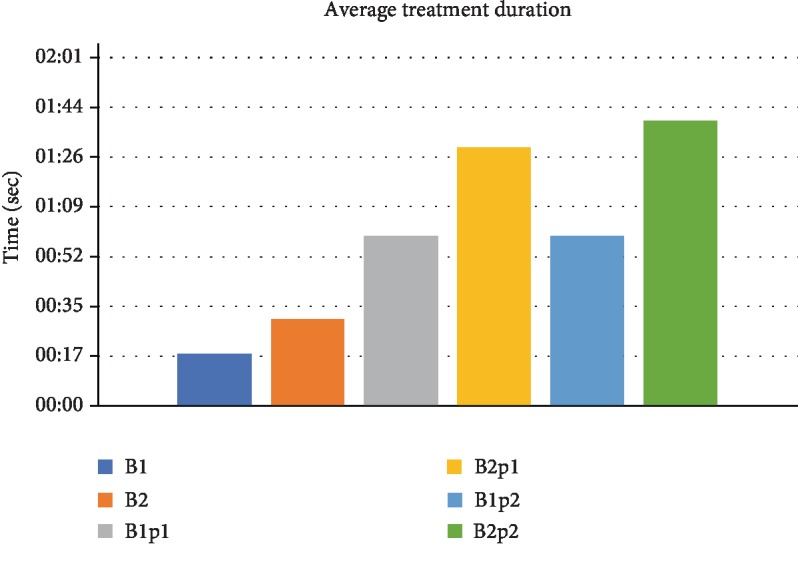
Average treatment duration (in seconds) for each RFE pattern.

The main treatment patterns evaluated were continuous versus pulsed mode. Half of the designated areas were treated once and the other half twice, as the whole procedure was carried out manually. Furthermore, in the pulsed mode, RFE activation was 1 second followed by a 1-second pause (Figure 1) or 2 seconds followed by a 2-second pause (Figure 1). The temperature was recorded simultaneously via the inserted sensor. The time needed for the different treatment groups is depicted in Figure 2.

### 2.2. Live/Dead and Caspase 3/7 Analyses

Directly after the treatment with RFE, the tibia plateaus were processed for live/dead staining and active caspase 3/7 detection and imaged using a confocal laser scanning microscope (CLSM, Nikon Eclipse E600, Kawasaki, Japan). A diamond waver blade (Bühler Säge) was used to cut 1.5 mm thin osteochondral sections for the staining procedures, and a bigger block was utilized for CLSM. For the live/dead staining, one section was incubated with 1.0 ml of phosphate-buffered saline (PBS) containing 2 *μ*m calcein-acetoxymethylester and 4 *μ*m ethidium homodimer-1 (EthD-1) for 30 minutes at room temperature. The specimens were mounted on the CLSM stage and evaluated at 4x magnification. The photomicrographs where then implemented for quantitate analysis by counting live or dead and caspase 3/7-positive cells with ImageJ software.

For detection of cell apoptosis by caspase (Cas) 3/7 analysis, osteochondral sections were incubated overnight in a cell culture incubator with 4 *μ*m Cas 3/7 green detection reagent (Molecular Probes, Thermofisher, Dreieich, Germany) diluted in DMEM low-glucose supplemented (Gibco, Thermofisher) with 10% Fetal Calf Serum (FCS) (PAN-Biotech, Aidenbach, Germany). Afterwards, specimens were rinsed with PBS and imaged as described above.

### 2.3. Quantitative Topographical CLSM Analysis

CLSM was used for quantitative analysis of cartilage surface roughness. The tibia plateau explants from the treated joint surfaces (consisting of cartilage with underlying bone tissue) with dimensions of approximately 1 cm^2^ area and 3 mm thickness were fixed overnight in a 4% formaldehyde solution in 0.1 M phosphate buffer further supplemented with 15% saturated picric acid solution and 0.1% Triton X-100. Following three times washing with PBS, the specimens were immersed in a 2% aqueous solution of tannic acid overnight and subsequently rinsed 6 hours with several changes of H_2_O followed by an overnight impregnation with 4% AgNO_3_ dissolved in H_2_O. The silver-stained specimens were rinsed and dehydrated in ascending concentrations of acetone, followed by substitution with 100% tert-butanol. Finally, specimens were placed in small aluminum dishes, frozen in liquid nitrogen, and vacuum-dried.

The deeply black color of the cartilage surface achieved by this technique is excellent for follow-up CLSM imaging. First, qualitative images of the surfaces were acquired. Second, for quantitative characterization of the surfaces, various roughness parameters were measured using the Olympus LEXT OLS 4000 3D CLSM (Olympus, Hamburg, Germany). The surface roughness was expressed by the Root-Mean-Square parameter (Sq). The region of interest of each image was set to 1281 *μ*m × 1279 *μ*m with 216x magnification and laser intensity of 50%. Six areas of 4 mm^2^ per sample were marked under light microscopy. If the measurement generated Sq > 35 *μ*m, a different second angle was measured. If Sq was >40, 4 different angles were measured; margins of each treated section served as a control for that section. Repeated measurements at different locations for each specimen were undertaken to establish statistical inference. In total, 46 areas per pattern were evaluated.

### 2.4. Statistics

Statistical analysis was performed using IBM SPSS Statistics 24.0 software for Windows (SPSS, Chicago, IL, USA). The roughness data was normally distributed (Kolmogorov-Smirnov Test) and a one-way ANOVA test was conducted. The Least Significance Difference Test was chosen as a post hoc test, to explore the difference between the treatment groups. Since the underlying data of the live/dead staining were not normally distributed, a nonparametric test was applied (Kruskal-Wallis Test). Values of *p* < 0.05 were considered statistically significant.

## 3. Results

### 3.1. Evaluation of Chondrocyte Survival

**Figure 3:**
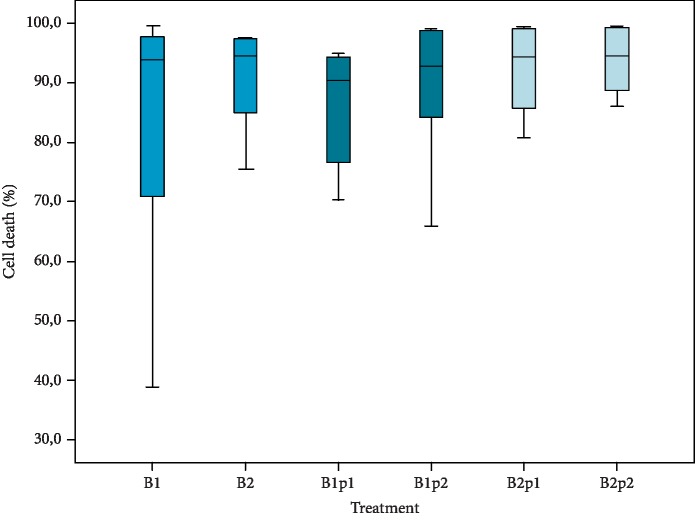
Rate of chondrocyte death with respect to RFE treatment pattern. Data is expressed as a percentage of dead cells to the total cell number. Box plot representing median ± I.Q.R. of *n* = 6.

**Figure 4:**
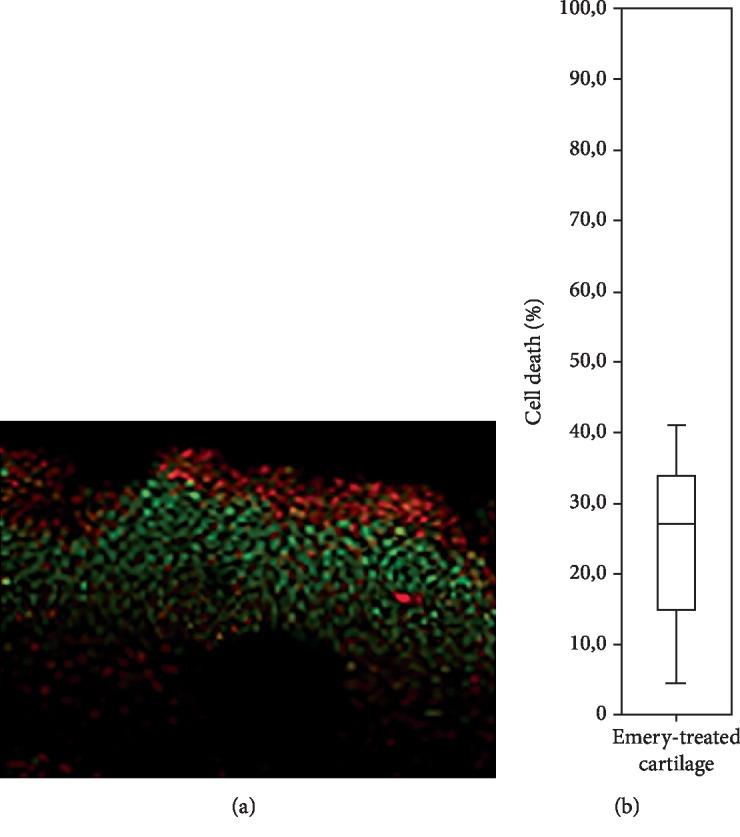
(a) Representative photomicrograph of live/dead stained articular cartilage treated with emery paper showing that cell death occurs in the superficial layer of cartilage. (b) Quantification of cell death rate in emery-treated cartilage samples. Box plot representing median ± I.Q.R. of *n* = 6.

The cell death rate for each group is shown in Figure 3 and Table 1. For continuous modes, B1 and B2, the median cell death in the cartilage layer was 93.9% and 94.5%, respectively. The lowest value was found for the B1p1 group with a median of 90.4% and followed by the B2p1 group with 92.9%. The highest death toll was in the B1p2 with 94.3% and in the B2p2 with 94.6%. Analysis of only emery-treated cartilage samples shows that cell death is primarily restricted to the superficial layer of cartilage with minimal cell death in the deep zones of the tissue. Quantitative analysis of this group demonstrates that there is a substantially lower cell death rate (mean: 25%) compared to samples subjected to RFE (Figure 4 and Table 1).

**Figure 5:**
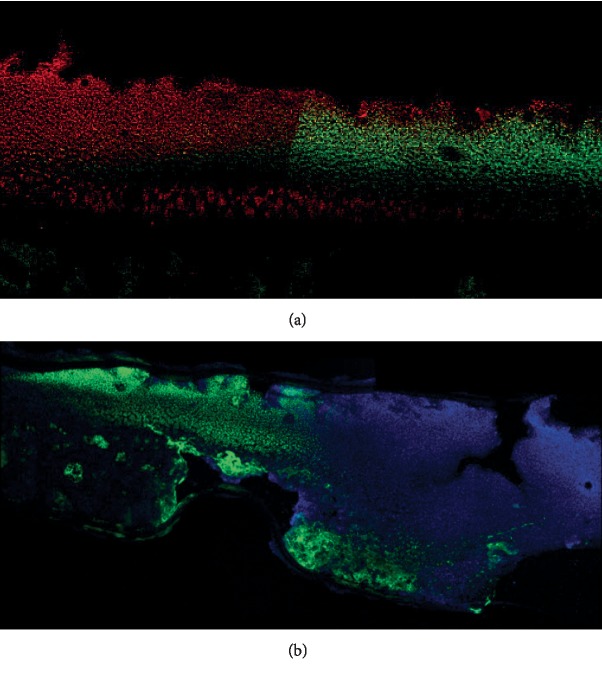
Photomicrographs of live/dead staining within the cartilage and underlying subchondral bone describing the (a) thermal penetration produced during treatment with B2 pattern and resultant cell viability with green dots indicating live chondrocytes, whilst red dots are dead chondrocytes. Representative image of caspase staining at (b) 24 hours post RFE treatment with blue dots showing nuclei of live chondrocytes whilst green dots labelled caspase-positive dead chondrocytes. Microscope magnification: 10x.

Statistically, there were no significant differences between the treatments (*p* = 0.744), although the death rate was higher, when two RFE passes were applied. These results were further validated by the apoptosis-specific caspase staining (Figure 5). In sum, our results demonstrated that all implemented RFE patterns and application modes caused profound cell death in the cartilage layer.

### 3.2. Quantitative Topographical Analysis

**Figure 6:**
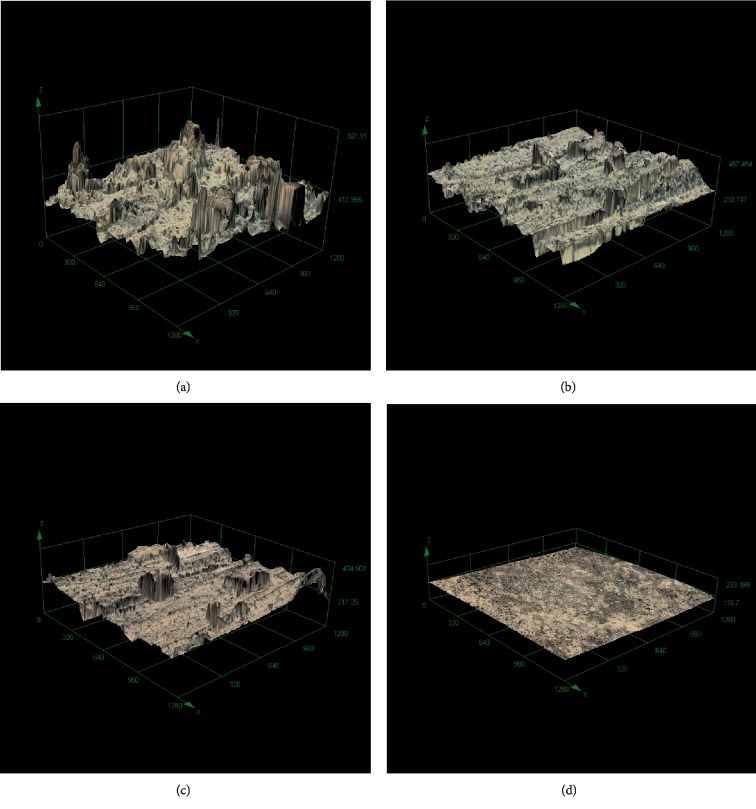
Three-dimensional CLMS images of (a) chondromalacic cartilage generated by emery paper (Sq = 58.4 *μ*m); (b) after continuous treatment B1, one pass (Sq = 37.7 *μ*m); (c) continuous treatment B2, two passes (Sq = 32.0 *μ*m); and (d) native cartilage (Sq = 2.7 *μ*m). Sq means surface roughness.

The surface roughness was expressed through the Root-Mean-Square Sq, which for native healthy cartilage is Sq = 3.8 ± 1.1 *μ*m (*n* = 24). Untreated osteoarthritic cartilage outerbridge grade III had a Sq = 42.6 ± 7.2 *μ*m (*n* = 27). The continuous treatment with one pass B1 showed a reduction of the roughness to Sq = 33.1 ± 8.5 *μ*m (*n* = 19) and with a second pass B2 to Sq = 31.3 ± 7.4 *μ*m (*n* = 21). The pulsed treatment B1p1 pattern with a 1-second pause and 1 pass reached a Sq = 34.1 ± 7.9 *μ*m (*n* = 18) and on the second pass B1p2 Sq = 28.0 ± 8.1 *μ*m (*n* = 15). A similar roughness was reached with a pulsed treatment pattern with a 2-second pause in the first pass B2p1 with Sq = 30.2 ± 8.6 *μ*m (*n* = 28), and the best result had this pattern on the second pass, B2p2 Sq = 27.3 ± 4.9 *μ*m (*n* = 26). We could find in all RFE treatment groups a statistically significant reduction of the cartilage surface roughness compared to the untreated osteoarthritic cartilage (*p* < 0.001). There was no statistical difference between the treatment patterns. Altogether, based on the Sq parameter, the best result was the B2p2 RFE-pulsed pattern with a 2-second pause and 2 passes.  Figure 6 shows representative three-dimensional images obtained by CSLM for native, OA, B1, and B2 treatment groups.

### 3.3. Time and Temperature Relationship

Regarding time-dependent temperature changes, for the B1 pattern, the maximum temperature was 34.1°C (*n* = 6) within a mean treatment time of 18 seconds. In the B2 group, an increased maximum temperature of 40.8°C (*n* = 6) was detected at an interval of 30 seconds.

**Figure 7:**
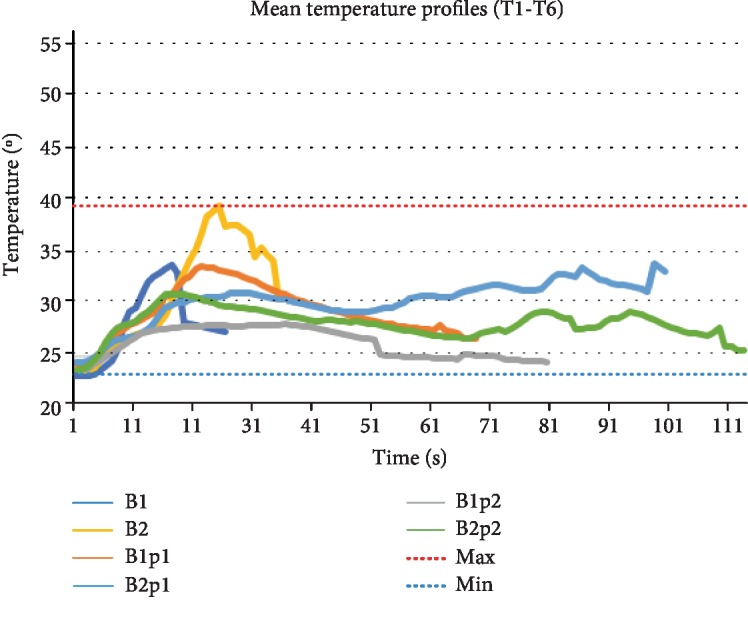
Time/temperature curve of the different RFE patterns.

Figure 7 shows that for both continuous RFE modes, a steep rise in the temperature, when compared to a steadier increase with a plateau-like phase for the RFE-pulsed mode. For this mode, the maximum temperature in the B1p1group was 34.4°C (*n* = 6), B1p2 28.7°C (*n* = 6), B2p1 34.2°C (*n* = 6), and B2p2 31.7°C (*n* = 6) (Table 1). In sum, apart from the B2 group, the maximum temperatures reached for the other groups were reasonable, suggesting that another parameter, independent of temperature, may trigger the increased cell death observed. It can be speculated that the cell death was not trigged by the produced heat but rather by the melting of the cartilage matrix and cells embedded within its structure. However, the exact mechanisms responsible for the cell death are to be clarified in future experiments.

## 4. Discussion

In our study, we investigated the smoothing effect of the cartilage surface with a bipolar RFE device. With quantitative topographical analysis, we found that the smoothing effect was dependent on the number of RFE passes over the cartilage surface. However, with increased smoothing, an augmentation in chondrocyte death up to 95% was detected.

In a previous study that evaluated different RFE devices, different grades of smoothness were achieved for the surface of the cartilage with variable chondrocyte cell death [3]. In another study, just 30 seconds of treatment was sufficient to achieve a smooth cartilage surface with cell death restricted to the subchondral layer. Here, a customized holder was used with very standardized RFE passes (weight, velocity). The authors applied one RFE pass of 5 seconds and already after 3 consecutive passes, a melting of the fibrillated cartilage was detected [13]. In our study, for the manual treatment of a cartilage area of 1 cm^2^, at least 17 seconds were needed to perform one continuous pattern with one pass (B1 pattern). Macroscopically, a smoothing of the cartilage surface was only visible after a second pass. Furthermore, in our experiments, already after one pass, the melting of the fibrillated cartilage was demonstrated by a decrease in the Sq value.

Kosy et al. postulated to move the RFE continuously and that only one pass should be enough to reach surface smoothening but with minimal thermal damage of the cells [18]. In contrast, our findings suggest that the continuous treatment with just one pass already triggers massive cell death (approx. 94%). Interestingly, only the pattern B1p1, had a lesser chondrocyte death than the other ones, although approximately only 10% of the cells in the cartilage layer survived. Moreover, our data suggests that the application time has a lesser impact than hypothesized [19], as well as the temperature increase being independent of the treatment pattern. In our experiment, the maximum temperature varied between 28.7 and 40.8 C°, which was measured in the subchondral bone in the middle of the section. It is hypothesized that the temperature affecting the above chondrocytes must be higher as shown in further experiments [4].

We also noted a tendency for the pulsed mode to have a lesser negative effect onto the chondrocytes. One explanation for the discrepancy is that Lu et al. conducted their experiment on osteochondral samples from knee replacements, where the cartilage layer has a thickness of 2-3 mm [13]. Our experiments were conducted on a thin cartilage layer similar to the small joint surface of the human wrist. We suggest that, in this case, the resident chondrocytes have a lower amount of surrounding territorial and interterritorial matrices that can protect them during RFE treatment. Thus, this approach is unsuitable for the regeneration of small joints.

In our study, the smoothing of the surface was also independent of the treatment pattern. However, a tendency towards roughness decreases after two RFE passes was detected. Still, the reached smoothing was far behind the values of native healthy cartilage. In sum, our findings are in line with two studies [13, 20] where following smoothing of the cartilage surface, a profound cell death was observed. Altogether, the postulation that RFE is a safe method for cartilage treatment [6] both in this study and by others means that this technique should be used with precautions for joints with a thin cartilage layer.

### 4.1. Limitation

One drawback of our study is that we tested only one bipolar RFE device. It has been suggested that there is a difference between device manufacturers, particularly in the metal used for ligation of the electrode tip and the insulation materials of the electrode wand that enable better chondrocyte survival [7, 21]. Furthermore, it has be shown that chondromalacic cartilage is more sensitive to higher temperatures than intact, healthy cartilage [22]. However, in our application modes, apart from the B2 group, the maximum temperatures reached for the other groups were reasonable, suggesting that a factor independent of the temperature could trigger the observed massive cell death.

## 5. Conclusion

All in all, there are multiple factors, which should be taken into account, when smoothing diseased cartilage with RFE in a clinical setting. In our study, based on the survival rate and apoptosis monitoring, a recommendation to use RFE for cartilage therapy cannot be given. We suggest that the baseline state of the cartilage subjected to treatment, RFE application mode, and duration, as well as the quality of the implemented RFE device, are critical but difficult to control in RFE regenerative therapy of small joints. Further research efforts are needed to standardize and control the technique, as well as to identify strategies to minimize the cell death rate to acceptable levels.

## Figures and Tables

**Table 1:** Table showing the relationship between temperature and cell death rate with respect to the treatment pattern.

Treatment pattern	Max temp (mean value)	Cell death (%)	Treatment time in sec. (mean value)	Temperature in °C (mean value)
B1	34.1	93.9%	00 : 18	28.0
B2	40.8	94.5%	00 : 30	31.2
B1p1	34.4	90.4%	00 : 59	29.4
B2p1	34.2	92.9%	00 : 59	30.1
B1p2	28.7	94.3%	01 : 30	26.8
B2p2	31.7	94.6%	01 : 39	28.4
Emery treated only	0	25.0%	0	0

## Data Availability

Most of the data used to support the findings of this study are included in the article, further data´s are available from the corresponding author upon request.
